# Intubated patient of malignant central airway obstruction due to advanced esophageal cancer on ventilator support treated with radiotherapy: a case report

**DOI:** 10.3332/ecancer.2025.1895

**Published:** 2025-04-17

**Authors:** Shubham Dokania, Ajay K Choubey, Shashank Tiwari, Prasenjit Nath, Jhansi Pattanaik, Sambit S Nanda, Ashutosh Mukherji, Satyajit Pradhan

**Affiliations:** 1 Department of Radiation Oncology, Mahamana Pandit MadanMohan Malaviya Cancer Centre, Varanasi 221005, Uttar Pradesh, India; 2 Department of Radiation Oncology, Mahamana Pandit MadanMohan Malaviya Cancer Centre (Under Homi Bhabha National Institute), Varanasi 221005, Uttar Pradesh, India; 3 Department of Anaesthesiology, Mahamana Pandit MadanMohan Malaviya Cancer Centre (Under Homi Bhabha National Institute), Varanasi 221005, Uttar Pradesh, India; a https://orcid.org/0009-0009-8200-3304

**Keywords:** esophagus, palliative radiotherapy, ventilator, airway obstruction

## Abstract

Malignancies, associated directly or indirectly with airways, can cause a fatal condition, called malignant central airway obstruction (MCAO). Both endoluminal and extraluminal MCAO are conventionally treated using bronchoscopy-mediated therapies, but in patients unsuitable for bronchoscopic interventions, there are very limited options, especially if the patient needs ventilator support due to the obstruction. Few patients of carcinoma lung with MCAO needing ventilator support have been treated using radiotherapy (RT), with appreciable results. Though multiple risks are associated with shifting such patients out of the intensive care unit, these steps had to be carried out when RT was deemed the only possible solution. In this rare case of carcinoma esophagus with MCAO needing ventilator support and treated with RT, we have shown how RT can be used to increase the possibility of extubation of the patient. Hence, respiratory relief and extubation were aimed and achieved with RT in this case.

## Introduction

Malignant central airway obstruction (MCAO) is an entity occurring in 75% of patients due to lung cancer, while the rest 25% of cases are attributable to the local or metastatic spread of a non-bronchogenic tumour [1]. The obstruction may be endoluminal, extraluminal or mixed, which decides the course of management. Purely endoluminal MCAO is treated with any modality directed at debulking the intraluminal disease, while extraluminal ones are conventionally managed by dilatation and stenting to relieve the external pressure. Radiotherapy (RT) is another feasible option in the form of intraluminal brachytherapy for endoluminal MCAO and high-dose external beam RT (EBRT) for extraluminal ones. A significant proportion of such patients need assisted ventilation, sometimes even invasive ones; hence, the option of EBRT is not considered, especially in low-resource settings. We present a case report of a patient with extraluminal MCAO due to progressive esophageal cancer treated successfully with high-dose EBRT, while she was on ventilator support.

## Case report

**Figure 1.**
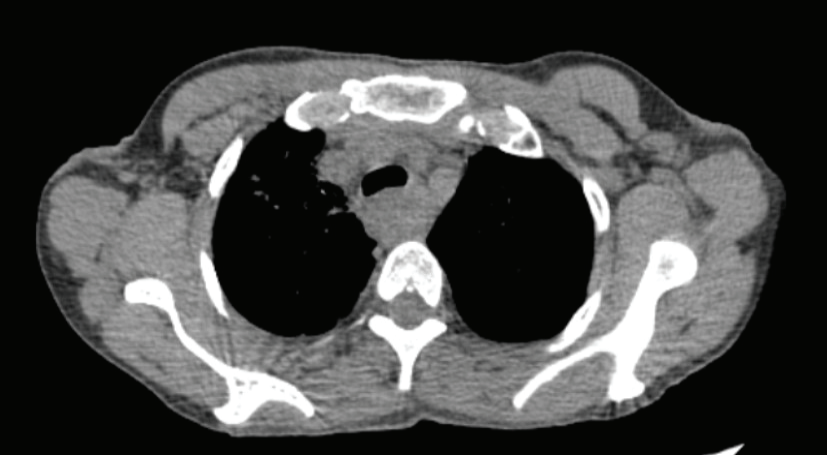
Axial CT scan images of the patient taken at the time of the baseline diagnostic scan.

A 48-year-old female, addicted to tobacco for 20 years, with no known comorbidities, presented to our hospital in April 2024 with the complaint of difficulty in swallowing solid foods and liquids intermittently for the last 6 months. Upper gastrointestinal endoscopy revealed a circumferential growth at around 20 cm from incisors with significant luminal narrowing, through which the scope could not be negotiated. Biopsy from the mass showed moderately differentiated squamous cell carcinoma. Fludeoxyglucose-18 (FDG) positron emission tomography (PET) computed tomography (CT) (F-18-FDG PET-CT) showed FDG avid circumferential wall thickening involving the cervical and upper third of the esophagus with a max thickness of 1.5 cm, SUVmax 14.88 and a craniocaudal length of 6.2 cm closely abutting the trachea, bilateral bronchi, descending aorta, azygous vein and adjacent vertebrae along with an FDG avid right supraclavicular node measuring 1.0 cm. The corresponding CT axial slice is shown in Figure 1.

The patient was diagnosed with carcinoma of the cervical and upper third of the esophagus, cT3N1 as per AJCC 8th edition classification, and was planned for definitive chemoradiotherapy (CTRT) to the primary tumour and involved nodes as per standard guidelines. The pulmonary function test (PFT) was within normal limits. RT planning contrast-enhanced CT scan was acquired using required positioning and immobilisation devices, with a 3 mm slice thickness. Post planning patient was called for starting radiation but the patient defaulted to treatment.

**Figure 2.**
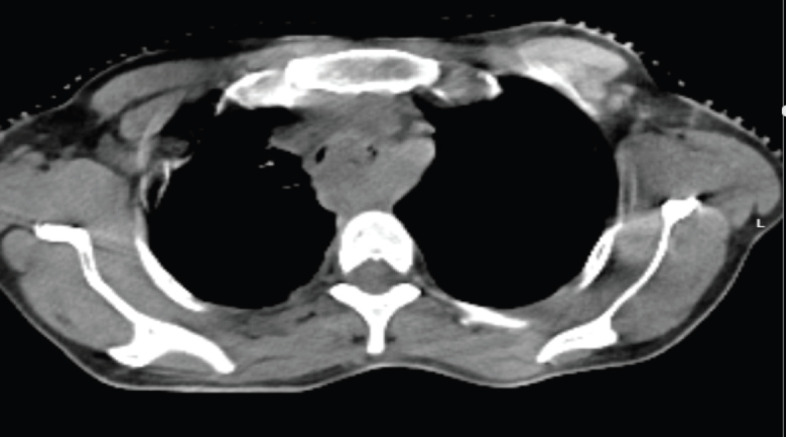
Axial CT when she presented with symptoms of dyspnea, showing MCAO.

She presented back to us after 2 months with aggravated symptoms of dysphagia to liquids also and intermittent respiratory obstruction suggestive of clinical progression. The patient was advised admission for supportive treatment and re-evaluation but refused. After 2 days, she landed in the emergency with acute respiratory discomfort and was admitted for evaluation and supportive treatment. CT thorax showed the esophageal mass almost completely obliterating the tracheal lumen above the carina, as shown in Figure 2. The patient started developing progressive dyspnea, getting worsened in the supine position (orthopnea) and had to be kept on oxygen support via a face mask in a propped-up position. Stenting was deferred due to the unavailability of a chest physician during that period and poor visibility beyond the obstruction. She was planned for high-dose RT to the primary tumour to relieve obstructive respiratory symptoms. An RT dose of 8 Gray in 2 fractions at 4 Gy/fraction, 3 days apart was to be delivered in the supine position with IV steroid support. However, while delivering the first fraction in the supine position, she started desaturating with the development of facial erythema so treatment had to be deferred. Due to her inability to lie down, she was planned for conventional 2-dimensional RT using anatomical landmark-based clinical marking on the patient’s body, and treatment portals were confirmed using portal imaging. The first 4 Gy fraction was delivered in a sitting position on the trolley using a single anterior field with the gantry rotated 90 degrees clockwise, under oxygen support. Given the anticipated edema post-RT, high-dose steroids were administered. Even after this prophylactic measure, her dyspnea worsened.

**Figure 3.**
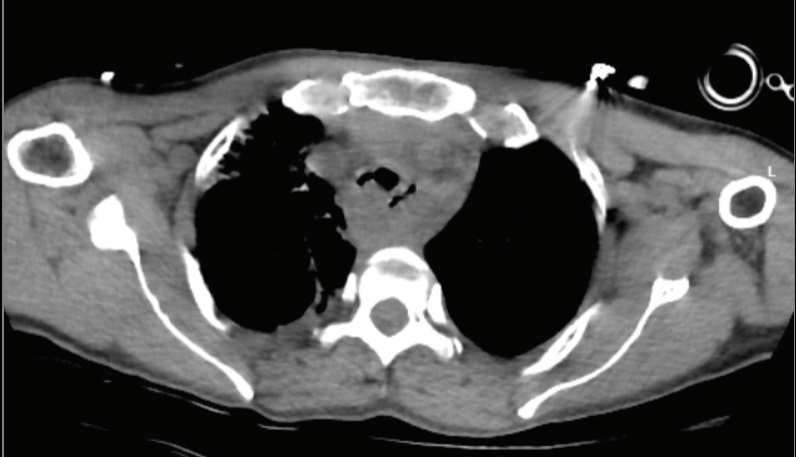
Response assessment scan done on day 4 of ICU stay-mild regression in airway obstruction.

After consultation with the Critical care team, she was shifted to the intensive care unit (ICU) for intubation for assisted ventilation. Under fiber-optic bronchoscopic guidance, a 7.0 French flexometallic tube was inserted through the right nostril and was started on mechanical ventilation with volume-limited assist control (VAC) mode, with a 40% fraction of inspired oxygen (FiO_2_), 6 mmHg positive end-expiratory pressure (PEEP) and tidal volume of 320 mL. The tube could not go beyond the tumour due to its limited length. She tolerated the procedure well. Thereafter, her condition gradually improved and was relieved of her dyspnea. She was started on IV antibiotics. On day 2 of the ICU stay, she was shifted to pressure support ventilation (PSV) mode and slowly weaned off the ventilator support under ABG monitoring. She was kept on a T-piece with 5 L per minute of oxygen support. Chest physiotherapy was being done along with incentive spirometry. Despite frequent suctioning, she had intermittent nasal endotracheal tube blockage attributable to her copious secretions and the small size of the tube, so the tube had to be replaced a total of four times between days 2 to 3 of the ICU stay. She maintained saturation on the t-piece for one and a half days but then started needing PSV mode intermittently. On day 4 of her ICU stay, she was shifted to PSV mode, with a 70% FiO_2_ and pressure support of 5 cm H_2_O. A response assessment non-contrast CT thorax was done, which showed mild regression in the extent of tracheal compression by esophageal mass along with secretions and aspirations, leading to basal atelectasis and consolidative changes in both lungs, as shown in Figure 3. On day 5, she was shifted back to VAC mode, with 40% FiO_2_, 6 mmHg PEEP and tidal volume of 320 mL, after ABG showed worsening of parameters. Sedation with fentanyl at 4 mL every hour was started.

**Figure 4.**
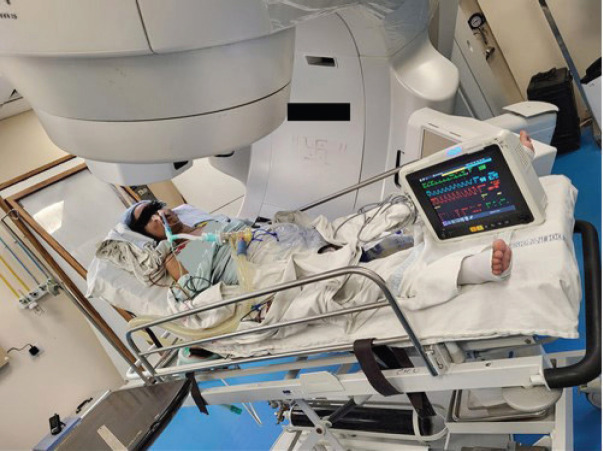
Patient on ventilator support and vital monitors, for a second high-dose RT fraction, in the treatment room.

**Figure 5.**
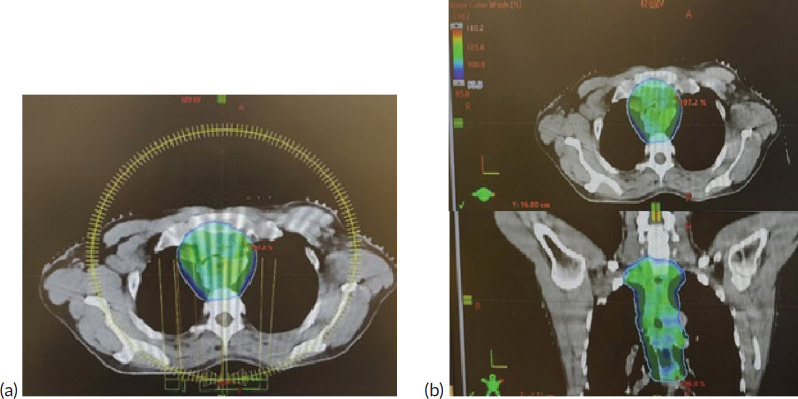
(a): VMAT plan with arcs used in the first consolidative RT plan. (b): 95% dose distribution achieved, as shown in axial and coronal views.

To prevent tube blockage and ensure proper suctioning, tracheostomy and flexometallic tube insertion were discussed but aborted as the likely benefit was going to be minimal due to obstruction below the planned tracheostomy site and only a proper anticancer treatment could give relief. After a thorough review of available logistics and equipment, the ICU and RT team agreed on transferring the patient on ventilator support from the ICU to the RT department for a second 4 Gy fraction of the planned high-dose RT for the MCAO. With proper coordination among the teams at various points in the transit, she was shifted out of the ICU on a portable ventilator and necessary monitors. The patient was not transferred from her ICU bed to the treatment couch. Her bed was made to orient along a 90-degree couch rotation. The bed was given an elevation of around 45 degrees and the gantry moved 30 degrees in the clockwise direction, as shown in Figure 4. After portal imaging and confirmation of a clinically marked single anterior portal, the second fraction of 4 Gy was delivered. She was safely taken back to the ICU after treatment and a high-dose steroid single dose was delivered as a prophylaxis against further worsening due to edema. On day 6, fentanyl sedation was stopped and antibiotics escalated to IV meropenem for raised total leukocute count, while she still needed ventilator support on VAC mode with 50% FiO_2_. On day 7, however, she was stepped down to continuous positive airway pressure mode, then weaned off ventilator support on a t-piece, and finally extubated the next day. Thereafter, she maintained saturation on a face mask with 4 L per minute oxygen support. Nondirected bronchoalveolar lavage flashed positive for multidrug-resistant *Klebsiella pneumoniae*, sensitive to Imipenem. She had already been on meropenem antibiotics since day 6 of ICU. On day 8, the patient was finally shifted to the ward from the ICU. She was off oxygen support in the ward the next day, without any difficulty in breathing although her absolute dysphagia persisted. One week after the 8 Gy in 2 fractions dose, when the patient was clinically and hemodynamically stable, she was planned for conventionally fractionated RT. First, 10 Gy in 5 daily fractions of 2 Gy each, followed by 34.2 Gy in 19 daily fractions of 1.8 Gy each (with reduced fields after 25.2 Gy to control dose to organs at risk) 5 days a week to a total equivalent dose in 2 Gy fractions (EQD2) of 60 Gy. Five cycles of concurrent chemotherapy of weekly Paclitaxel (75 mg/m^2^ i.v.) and Carboplatin (Area under curve 2 i.v.) were administered, with weekly monitoring of blood parameters. The treatment fields (arcs) and dose distribution of the first consolidative RT plan have been illustrated in Figure 5. The consolidative treatment lasted for around 6 weeks, from 12th August 2024 to 24th September 2024, with planned gaps in between as per the patient’s symptoms.

**Figure 6.**
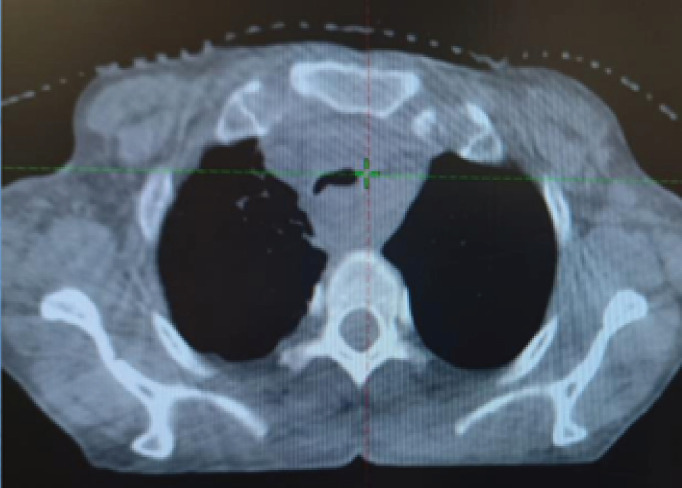
CBCT at first consolidative RT fraction showing regression in the tracheal compression.

**Figure 7.**
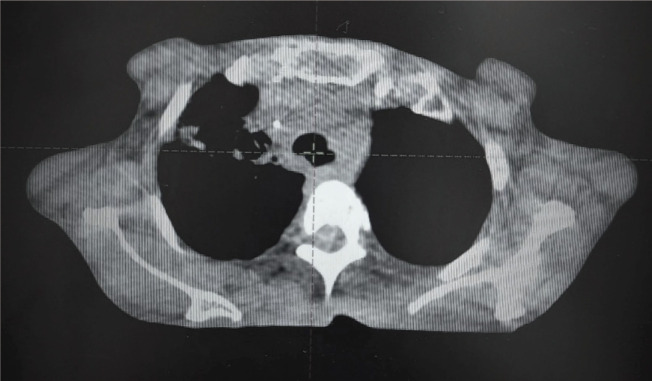
CBCT at last RT fraction showing complete resolution of tracheal compression.

The cone beam CT (CBCT) images taken on the first consolidative RT fraction, as shown in Figure 6, and that on the last fraction of consolidative RT, as shown in Figure 7, show progressive resolution of tracheal compression. At the 1-week follow-up, she had no respiratory discomfort, but was on percutaneous endoscopic gastrostomy-based nutritional support, due to persistent dysphagia. The patient was reviewed again in December 2024, and she had no respiratory discomfort. She was wheelchair-bound and on feeding jejunostomy-based nutrition support. She was advised to continue supportive treatment and review again after 3 months with cross-section imaging for response assessment.

## Discussion

A common and potentially fatal side effect of malignancies, associated directly or indirectly with airways, is MCAO, which is characterised by a reduction of more than 50% of a proximal airway calibre [2]. This results in dyspnea, orthopnea and infections secondary to obstruction, with the patient soon landing up in the ICU. The definitive treatment of the malignancy gets delayed and complicated, for example, the ensuing atelectasis may cause difficulty in target volume delineation for lung cancers. The causes of endoluminal MCAO include primary lung carcinoma, metastases to the airways from primaries in the kidney, colon, sarcoma and airway carcinoids. Extraluminal MCAO is attributed to the local progression of carcinomas of the esophagus, thyroid and mediastinal tumours.

The conventional management of MCAO is therapeutic bronchoscopy, directed at tumour debulking via laser therapy and intraluminal brachytherapy for endoluminal MCAO. Extraluminal MCAO necessitates bronchoscopy-mediated dilatation and stenting to overcome any centripetal force. In their retrospective study of 12 intubated and mechanically ventilated patients of inoperable non-small-cell lung cancers, Murgu *et al* [2] showed that immediate extubation and weaning off ventilator support was possible in 75% of patients post bronchoscopic interventions. Similarly, Daigmorte *et al* [1] showed that therapeutic bronchoscopy for patients with airway metastases from non-bronchogenic cancers led to rapid symptomatic improvement and prolonged survival, though only 18% of their cohort required ventilator support. As far as extraluminal MCAO is concerned, the dramatic and immediate resolution of symptoms with bronchoscopy and stenting has been shown by Chan *et al* [3] though their cohort included only 36% of patients on mechanical ventilation.

However, patients with normal airways beyond the obstruction invisible on bronchoscopy, those with short expected life expectancy despite the intervention or those with advanced cancer, restricted mobility and showing few symptoms, are considered ineligible for bronchoscopic interventions [4]. The reason our patient was not taken for bronchoscopic intervention was poor visibility beyond the obstruction and unavailability of a chest physician during this period, and hence, we had to resort to high-dose RT initially as a feasible option. Guidelines do exist for the role of intraluminal brachytherapy in patients with proximal endoluminal MCAO [5]. However, the circumstances are quite different in intubated patients on mechanical ventilation. Louie *et al* [6] in their retrospective review of 26 patients of MCAO due to primary lung cancers (96%), showed the benefit of RT while on ventilator support [6]. With the dose delivered over a short course, ranging from 12.5 to 22 Gy (median dose- 20 Gy) 27% were extubated between 4 and 22 days after the start of RT. All the patients were successfully discharged from the ICU and 23% were even sent back home. Although the median survival was poor (10.8 days, range 0 to 113 months), the utility of RT in ventilated patients was well-established due to successful extubation in more than a quarter of patients. Also, a higher RT dose was predictive of better overall survival. Similarly, Ghiam *et al* [7] retrospectively studied 22 intubated patients of MCAO, mainly due to carcinoma lung, who were treated with any number of RT fractions while on mechanical ventilation support. With the median dose of 20 Gy (range 0.3 to 70 Gy) delivered over 1–35 fractions (median 6), 13 patients (59%) were successfully extubated and 11 (50%) could be discharged from the hospital. Thus, high-dose RT is a good option in ventilated patients with MCAO with difficult extubation or before the situation worsens leading to prolonged mechanical ventilation and aggravating signs and symptoms. This was very well exemplified in our case. A tremendous change in the patient’s condition was witnessed before and after the second fraction of high-dose RT. A patient struggling for her life and breath, on ventilator support, was weaned off just 2 days after RT, shifted out from the ICU to the ward and maintained saturation on room air just after 4 days of RT. The improvement in quality of life can be estimated from the fact that she was fit enough for receiving chemotherapy concurrently with RT, just a few days later.

However, transporting an intubated patient on ventilator support out of the ICU carries its risks. Voigt *et al* [8] showed that ICU patients transported out for any reason used vasopressors more frequently, required mechanical ventilation, had longer ICU and hospital stays and had higher hospital death rates than non-transported patients. Similarly, Beckmann *et al* [9] showed that out of 176 ICU patients transported out for diagnostic or therapeutic purposes, 31% had an adverse event. Other logistic issues including those with monitoring, oxygen supplies, travel ventilators, intubation equipment and battery/power supply also need to be considered before planning to shift the patient out of the ICU. Another aspect of high-dose RT to the airway region is the risk of life-threatening pulmonary and cardiac complications, however careful balance must be made between the degree of anticipated improvement and the degree of toxicity, as acute toxicities can aggravate the condition of a ventilated patient. A baseline PFT can be useful in making such decisions [10]. In our case also, the decision to treat an intubated patient on mechanical ventilation on an RT machine was not an easy one. Multiple rounds of discussions were done, among the RT team and ICU team. The availability of oxygen supply inside the treatment room and other infrastructural requirements were verified by the ICU team. Deeming RT to be the only definitive treatment option for the mass impinging on the trachea, this tough step was the need of the hour and was taken with utmost caution.

Other anticipated complications of high-dose RT to the esophagus mass were tracheo-esophageal fistula (TEF) and esophageal stricture. The incidence of TEF post-RT for esophageal cancer is 10.4%–13.9%, with associated poor prognosis [11]. The predisposing risk factors for the same include concomitant use of chemotherapy, young age and total circumferential lesion, all of which were present in our case [12]. Its risk may be mitigated by administering induction chemotherapy first, followed by RT or CTRT (though this was not feasible in our case) or lower total dose or dose per fraction. The next complication, esophageal stricture, is seen rarely under 30 Gy; however, the incidence rates at 50 and 60 Gy are 2% and 15%, respectively [13]. This may further worsen the quality of life of the patient

Another oncological management of intubated patients of highly chemosensitive tumours like small-cell lung cancer (SCLC) causing MCAO is administering chemotherapy [14]. Jennens *et al* [15] in their small retrospective study showed that two out of five SCLC patients intubated due to MAO when treated with chemotherapy, could be successfully extubated and were alive 7 months later. There is even a case report of bronchoscopic intratumoural instillation of chemotherapy for tracheal tumours, with successful shrinkage of the tumour [16]. Chemotherapy again will have its associated toxicities, which may be detrimental to the already worse condition of an intubated patient. This option was not available, though, in our case, because the patient was already harbouring a blood/chest infection, and was on higher antibiotics for the same.

After the airway obstruction component of the disease has been dealt with, the definitive treatment of the malignancy per se, was also equally important, to avoid repeated MCAO. From an esophageal cancer point of view, RT effectively palliates dysphagia in 34% to 48% of inoperable patients, with serious complications only in 2% of patients [17]. For our case, we started her on definitive treatment of the esophageal disease, i.e., conventionally fractionated RT, just after 3 days of shifting to the ward, to decrease the chances of tumour repopulation. Once the infective component settled, we also planned for concurrent chemotherapy to further aid in the regression of the disease. There is no reported case of esophageal cancer patient causing MCAO and getting symptomatically relieved post high-dose RT, while on ventilator support, and ours is most likely the first of its kind.

## Conclusion

For locally advanced esophageal cancer-causing MCAO, RT is a good option for symptomatic relief to the patient. This holds even when the patient is intubated and is on mechanical ventilation, to wean them off the ventilator. However, transferring MCAO patients on ventilatory support out of the ICU and irradiating them should be done after thorough discussion and ensuring the availability of necessary logistics. Though delivering RT for symptomatic relief, while the patient was on mechanical ventilation worked pretty well in our case, existing literature shows that only 27% to 59% of MCAO patients could be extubated and benefitted.

## Conflicts of interest

There are no conflicts of interest.

## Funding

No funds were received for writing this manuscript.

## Informed consent

Informed consent has been obtained from the patient.

## Author contributions

Conception and design- Ajay K Choubey, Shubham Dokania, Sambit S Nanda, Ashutosh Mukherji, Satyajit Pradhan.

Drafting the work and reviewing- Shubham Dokania, Ajay K Choubey, Shashank Tiwari, Prasenjit Nath, Jhansi Pattanaik, Sambit S Nanda.

Final approval- Ajay K Choubey, Ashutosh Mukherji, Satyajit Pradhan.
